# Isolated Abducens Nerve Palsy as the Sentinel Sign of Clival Metastasis in High-Grade Neuroendocrine Carcinoma: A Case Report

**DOI:** 10.7759/cureus.82445

**Published:** 2025-04-17

**Authors:** Mehdi Mounir, Kaye Ndi Paola, Ilias Bennouna, Philomene Lavis, Anais Eskenazi

**Affiliations:** 1 Department of Internal Medicine, Centre Hospitalier Interrégional Edith Cavell (CHIREC) Braine l'Alleud, Bruxelles, BEL; 2 Department of Radiology, Centre Hospitalier Interrégional Edith Cavell (CHIREC) Braine l'Alleud, Bruxelles, BEL; 3 Inter-regional University Center of Expertise in Hospital Pathological Anatomy, Curepath, Jumet, BEL

**Keywords:** clival metastasis, isolated abducens nerve palsy, neuroendocrine carcinoma (nec), occult primary tumor, skull-base metastasis

## Abstract

We report a rare case of clival metastasis from a high-grade neuroendocrine carcinoma (NEC) of unknown primary origin. A 71-year-old man, previously cured of prostate adenocarcinoma, presented with abdominal pain leading to the diagnosis of metastatic NEC (Ki67 >90%) involving the liver and bones. After three lines of chemotherapy and immunotherapy, he developed sudden diplopia and right abducens nerve palsy. A brain contrast-enhanced MRI revealed a clival mass compressing the sixth cranial nerve, which was undetectable on prior imaging. The patient received palliative radiotherapy and corticosteroids without a significant clinical response. While neuroendocrine neoplasms (NENs) frequently metastasize to visceral organs, clival involvement is exceptionally rare, particularly as a delayed complication. To our knowledge, this is the first reported case of NEC with clival metastasis, emphasizing the tumor’s aggressiveness. This case underscores the critical role of advanced neuroimaging in detecting atypical presentations in patients with new neurological deficits, even under systemic therapy, and highlights the challenges of managing skull base metastases in aggressive malignancies. Radiotherapy may stabilize symptoms, but functional recovery remains limited in cases of prolonged nerve compression.

## Introduction

Neuroendocrine neoplasms (NENs) are rare abnormal growths that originate from widely distributed cells within the neuroendocrine system. Histologically categorized into two distinct entities - well-differentiated neuroendocrine tumors (NETs) and poorly differentiated neuroendocrine carcinomas (NECs) - their classification relies on proliferative activity and morphological criteria. Key diagnostic markers include the mitotic count (number of dividing cells per 2 mm²) and the Ki-67 index, a proliferation marker correlating with cellular division rates [1].

NETs are graded as G1 (Ki-67 ≤3%, mitotic count <2/2 mm²), G2 (Ki-67 3-20%, mitotic count 2-20/2 mm²), or G3 (Ki-67 >20%, mitotic count >20/2 mm²), typically exhibiting indolent growth, organoid architecture, and hormone-secreting potential. By contrast, NECs are high-grade malignancies (Ki-67 >20%, often exceeding 55%) characterized by marked cytological atypia, necrosis, and aggressive behavior, with early metastases and a median survival under 12 months despite therapy [1,2].

While the liver, lungs, bones, and brain are common sites of dissemination, metastases to the skull base, particularly the clivus, are exceedingly rare [3,4]. Clival metastases are more frequently associated with cancers of the prostate, gastrointestinal tract, lung, and kidney, with only a handful of cases reported in the context of NENs [5-7].

To our knowledge, this represents the first documented instance of clival metastasis arising from NEC of unknown primary origin.

## Case presentation

**Figure 1:**
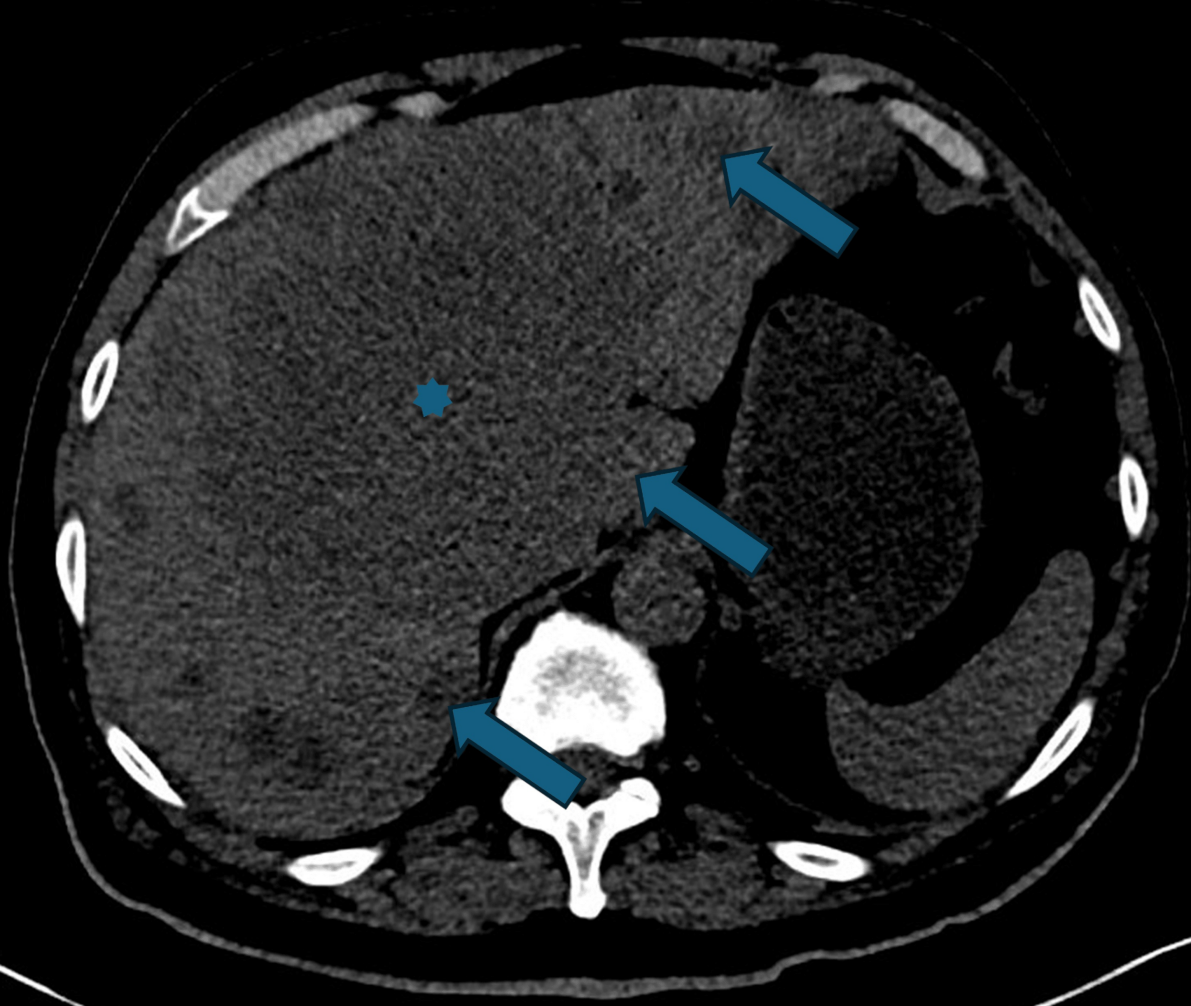
Axial non contrast enhanced abdominal CT scan Multiple liver metastasis (arrows). Central hepatic mass (star)

**Figure 2:**
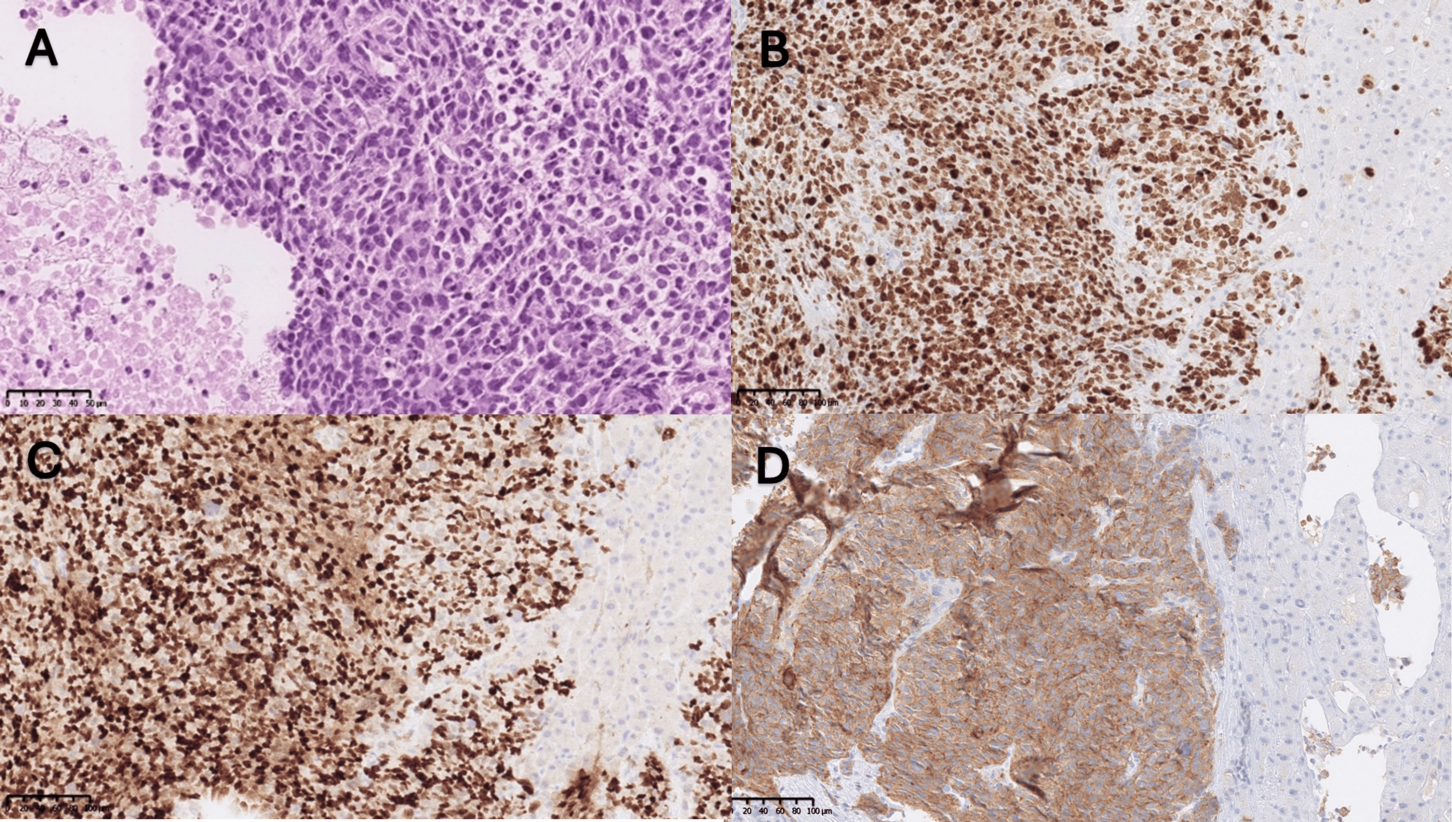
Histopathological and Immunohistochemical Features of the Hepatic Lesion (A) Hepatic parenchyma infiltrated by a poorly differentiated neoplastic process with areas of necrosis (H&E 20x). (B) Very high proliferation index (>90%) (Ki67 20x). (C) Diffuse and intense nuclear expression in neoplastic proliferation (INSM1 20x). (D) Diffuse moderate-intensity positivity (Synaptophysin 20x).

A 71-year-old man, with a history of successfully treated prostate adenocarcinoma (Gleason score 9) in 2021, undetectable PSA since treatment, presented in August 2023 with abdominal pain and weight loss. An abdominal CT scan revealed diffuse metastatic infiltration of the liver parenchyma, including a large 14 cm central hepatic mass (Figure 1). A liver biopsy confirmed high-grade small-cell NEC (Ki67 >90%, INSM1 +, synaptophysin +), with no adenocarcinoma component (Figure 2A-2D). A PET-CT staging workup identified hepatic and skeletal metastases, along with a non-contributory thyroid nodule.

The patient received first-line chemotherapy with carboplatin-etoposide (eight cycles), achieving a partial response marked by a significant decrease in AFP levels (from 39,775 to 160 µg/L, N < 7 µg/L) and partial regression in both size and number of hepatic metastases on follow-up thoraco-abdominal CT imaging. In the absence of a validated specific tumor marker for high-grade NECs, AFP - despite its lack of specificity - was used as a surrogate biomarker for disease monitoring, given its markedly elevated baseline level and dynamic correlation with the tumor’s clinical course.

**Figure 3:**
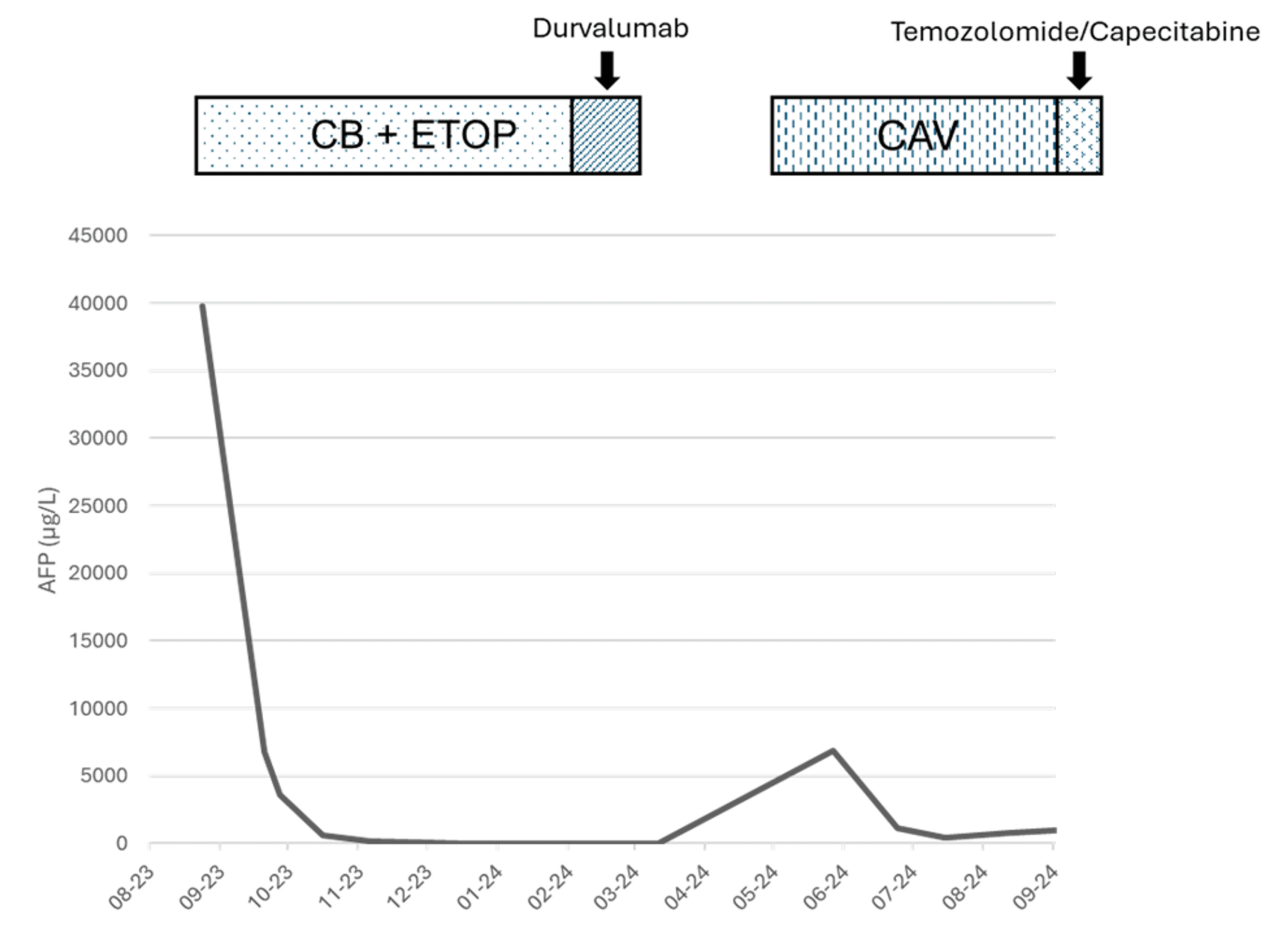
History of neuroendocrine carcinoma (NEC) treatment AFP: alpha-fetoprotein, CB: carboplatine, ETOP: etoposide, CAV: cyclophosphamide-doxorubicine-vincristine

In February 2024, immunotherapy with durvalumab was initiated but discontinued due to autoimmune myocarditis. Three months after treatment interruption, an abdominal CT scan revealed tumor progression, with AFP levels rising to 1136 µg/L. In June 2024, a second-line regimen of cyclophosphamide-doxorubicin-vincristine was started, followed by a third-line therapy with temozolomide-capecitabine in September 2024 due to further hepatic progression (AFP 1553 µg/L) (Figure 3).

**Figure 4:**
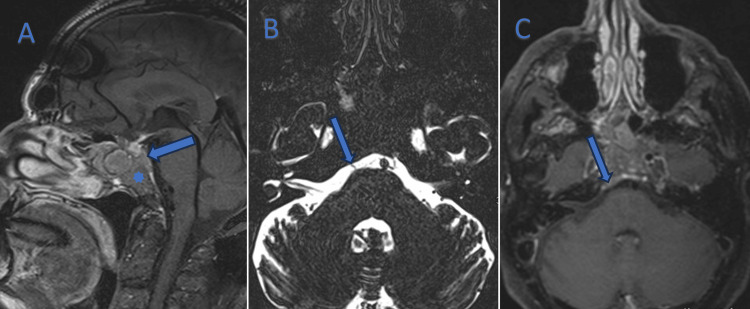
Brain MRI Sagittal dark blood contrast-enhanced (A): clival involvement (star). Notice the meningeal pre-clival tumor deposit (arrow). Axial space T2WI (B): right cisternal portion of the abducens nerve before entering Dorello’s canal (arrow). Axial dark blood contrast-enhanced (C): meningeal pre-clival tumor deposit (arrow) at the same level entry of the right abducens nerve in Dorello’s canal (see B).

In July 2024, 11 months after the initial diagnosis, the patient developed sudden horizontal diplopia and right exophthalmos. Neurological examination revealed complete loss of right eye abduction, with no other deficits. Brain MRI identified a 25 mm destructive clival lesion with retroclival epidural extension and invasion of the right cavernous sinus, compressing the abducens nerve (CN VI) within Dorello’s canal (Figure 4A-4C), which was undetectable on prior imaging six months earlier. Intravenous corticosteroids (methylprednisolone 1 mg/kg/day) and stereotactic radiotherapy transiently stabilized symptoms.

## Discussion

Clival tumors are exceptionally rare, representing 0.1-0.4% of all intracranial tumors [5]. Metastatic involvement of the clivus is even rarer, accounting for fewer than 1% of skull-base metastases, and is typically associated with primaries such as the prostate, gastrointestinal tract, lung, and kidney [5]. NECs, particularly those of unknown primary origin, are seldom implicated in clival metastases, making this case, to our best knowledge, a unique contribution to the literature.

The anatomical vulnerability of the abducens nerve (CN VI) within Dorello’s canal, a bony structure traversing the clivus, explains why a majority of clival metastases present with isolated CN VI palsy [5]. In our patient, acute diplopia and loss of right eye abduction were direct consequences of tumor compression at this site, aligning with the classical clinical presentation described in prior studies. This case underscores the importance of neuroimaging - specifically contrast-enhanced MRI - for detecting subtle clival lesions, as non-contrast imaging failed to identify the metastasis initially.

The diagnostic challenge of clival lesions is compounded by their broad differential, which includes chordomas, chondrosarcomas, and meningiomas [8]. In this case, imaging features such as aggressive bone destruction, retroclival epidural extension, and multifocal invasion argued against benign entities (e.g., meningioma) or slow-growing tumors (e.g., chordoma), which typically lack such rapid progression. Immunohistochemical (IHC) analysis of the hepatic lesion (INSM1+, synaptophysin+; TTF1-, NKX3.1-, SOX10-) further confirmed NEC and excluded alternative etiologies such as lung adenocarcinoma (TTF1-), prostatic recurrence (NKX3.1- despite the patient’s history), and melanoma (SOX10-). Nevertheless, the primary tumor remained elusive despite extensive investigations.

Therapeutic considerations

Therapeutic options for clival metastases remain limited and context-dependent. According to the systematic review by Carretta et al., out of 58 documented cases of clival metastases, only 39 patients underwent surgical intervention. Surgical management was typically reserved for specific cases requiring histopathological confirmation or presenting with significant neurological symptoms necessitating decompression [7]. However, most of these cases occur in advanced-stage disease, where surgery is rarely indicated. In such instances, palliative radiotherapy (RT) is often prioritized, particularly for fragile patients or those with limited oncological prospects. Studies have shown that RT can lead to improvements in symptoms such as headaches and diplopia [7,9].

When surgery is considered, the endoscopic endonasal approach (EEA) is considered the optimal surgical technique for clival lesions, offering superior visualization of the skull base, minimal invasiveness, and reduced morbidity compared to transcranial methods. While EEA enhances local tumor control and alleviates critical symptoms like vision loss, its risks, including cerebrospinal fluid leakage and meningitis, must be weighed against equivocal survival benefits. In aggressive malignancies such as NECs, where prognosis is predominantly governed by systemic progression, surgical intervention is generally reserved for scenarios where imminent functional deficits (e.g., vision loss) demand urgent decompression. For this patient, the infiltrative nature of the clival lesion, multifocal metastases, and rapid clinical decline rendered surgery inadvisable, underscoring the prioritization of systemic disease management in advanced NECs [10-12]. 

Recent studies highlight gamma knife radiosurgery (GKRS) as a safe and effective alternative for clival metastases, particularly for smaller lesions or patients with prior irradiation, achieving excellent tumor control rates [13].

## Conclusions

This case illustrates the aggressive biology of NECs and their capacity for atypical, delayed metastases. To our knowledge, this represents the first documented instance of clival metastasis arising from a high-grade NEC of unknown primary origin, underscoring the tumor’s potential for unpredictable dissemination. Systemic treatment remains challenging due to the rapid development of chemoresistance and the absence of any established second-line treatment options. While radiotherapy may transiently alleviate symptoms and surgery is rarely feasible in advanced disease, neurological recovery remains elusive, emphasizing the need for early detection and innovative therapeutic strategies. Clinicians should maintain a high index of suspicion for skull base involvement in NEC patients with unexplained cranial nerve deficits, even in the absence of prior neurological symptoms. Looking ahead, future management could benefit from personalized therapeutic approaches and greater inclusion of patients in clinical trials to improve outcomes for these rare, aggressive malignancies. Advanced neuroimaging and multidisciplinary collaboration are critical to address the challenges posed by these tumors, balancing symptom control with the urgent need for biologically driven therapies.
